# Placenta-specific novel splice variants of Rho GDP dissociation inhibitor β are highly expressed in cancerous cells

**DOI:** 10.1186/1756-0500-5-666

**Published:** 2012-12-03

**Authors:** Keiichi Hatakeyama, Yorikane Fukuda, Keiichi Ohshima, Masanori Terashima, Ken Yamaguchi, Tohru Mochizuki

**Affiliations:** 1 Medical Genetics Division, Shizuoka Cancer Center Research Institute, 1007 Shimonagakubo, Nagaizumi-cho, Sunto-gun, Shizuoka, 411-8777, Japan; 2 Gastric Surgery Division, Shizuoka Cancer Center Hospital, 1007 Shimonagakubo, Nagaizumi-cho, Sunto-gun, Shizuoka, 411-8777, Japan; 3 Shizuoka Cancer Center Hospital and Research Institute, 1007 Shimonagakubo, Nagaizumi-cho, Sunto-gun, Shizuoka, 411-8777, Japan; 4 Present address: G&G Science, 4-1-1 Misato, Matsukawa-machi, Fukusima, 960-1242, Japan

**Keywords:** Alternative splicing, ARHGDIB, Biomarker, Metastasis, Splice variant

## Abstract

**Background:**

Alternative splicing of pre-mRNA transcripts not only plays a role in normal molecular processes but is also associated with cancer development. While normal transcripts are ubiquitously expressed in normal tissues, splice variants created through abnormal alternative splicing events are often expressed in cancer cells. Although the Rho GDP dissociation inhibitor β (*ARHGDIB*) gene has been found to be ubiquitously expressed in normal tissues and involved in cancer development, the presence of splice variants of *ARHGDIB* has not yet been investigated.

**Results:**

Validation analysis for the presence of and exon structures of splice variants of *ARHGDIB*, performed using reverse-transcriptase polymerase chain reaction and DNA sequencing, successfully identified novel splice variants of *ARHGDIB*, that is, 6a, 6b, and 6c, in colon, pancreas, stomach, and breast cancer cell lines. Quantitative real-time polymerase chain reaction analysis showed that these variants were also highly expressed in normal placental tissue but not in other types of normal tissue.

**Conclusions:**

Expression of ARHGDIB variants 6a, 6b, and 6c appears to be restricted to cancer cells and normal placental tissue, suggesting that these variants possess cancer-specific functions and, as such, are potential cancer-related biomarkers.

## Background

Alternative splicing of pre-mRNA transcripts is an important process by which genomic complexity is generated from the small number of genes currently estimated to be present within the human genome sequence. Alternative splicing not only plays a role in normal molecular processes but is also associated with cancer development [1-5]. While normal transcripts are ubiquitously expressed in normal tissues [6,7], splice variants created through abnormal alternative splicing events are often expressed in cancer cells. When the different expression patterns of a splice variant and the normal transcript on the same gene can be detected, the variant is considered a potential biomarker.

The Rho GDP dissociation inhibitor β gene, commonly referred to as *ARHGDIB* but also known as LyGDI, GDI-D4, RhoGDI2, or RhoGDIβ, inhibits dissociation of GDP from Rho family GTPases. *ARHGDIB* has been reported to be involved in either the progression or suppression of metastasis [8-17] and is also ubiquitously expressed in normal tissues [11]. Despite these recent findings, the presence of splice variants of *ARHGDIB* in cancerous cell lines has not yet been investigated. Employing validation analysis using RT-PCR and DNA sequencing, this study aimed to fill this research gap by investigating alternative splicing events in cancer cell lines and normal tissues to identify splice variants of *ARHGDIB*. The analysis showed the presence of 3 novel splice variants of the *ARHGDIB* gene, and their expression was observed only in cancer cell lines and normal placenta. However, no protein isoforms were translated from these variants since their protein-coding regions were identical.

## Methods

### Cell lines and culture conditions

The cell lines used in this study are listed in Additional file 1: Table S1. The MKN45P cell lines [18] were kindly provided by Dr. Yutaka Yonemura and Dr. Yoshio Endo. These cell lines were cultured and maintained in RPMI 1640, DMEM/F12, or DMEM medium (Sigma-Aldrich, St. Louis, MO, USA) supplemented with 5–10% foetal bovine serum (FBS; Invitrogen, Carlsbad, CA, USA), glutamine (0.3 mg/ml), penicillin (100 unit/ml), and streptomycin (0.1 mg/ml), in a humidified 5% CO_2_ incubator. Protein expression and mRNA analyses were performed using cells found to have 70–90% confluency and greater than 95% viability, as determined using trypan blue staining.

### RNA extraction and cDNA synthesis

After total RNA had been extracted from each pellet using the RNeasy Plus Mini Kit (Qiagen, Hilden, Germany), RNA concentration was determined using a NanoDrop spectrophotometer (Thermo Fisher Scientific, Waltham, MA, USA), and total RNA quality was then confirmed using the Agilent 2100 Bioanalyzer (Agilent Technologies, Santa Clara, CA, USA). The purified total RNA from cancer cell lines and total RNA derived from normal tissues (Clontech, Mountain View, CA, USA) were then reverse-transcribed using ThermoScript Reverse Transcriptase (Invitrogen) and oligo(dT)_20_ primers in accordance with the manufacturer’s instructions. The normal tissues that were purchased are listed in Additional file 1: Table S2.

### Reverse-transcriptase PCR

Screening of cDNA for different splice variants of ARHGDIB was performed using the intron-spanning exonic primers listed in Table 1. PCR amplicons were designed to detect the splice variants and the transcript registered in GenBank. The cDNA synthesized was amplified using LA Taq polymerase (Takara Bio, Shiga, Japan) with 35 PCR cycles of 95°C for 30 sec, 51°C for 30 sec, and 68°C for 60 sec.

**Table 1:** DNA sequences of RT-PCR, quantitative RT-PCR, and DNA sequencing primers

**Target**	**Sense primer (location)**	**Antisense primer (location)**
RT-PCR and DNA sequencing		
NM_001175	ACTCAGAAGTCAGAGTTGAGAGAC (exon 1)	AGAGAATTCTTCCAGGTGGCAAGG (exon 6/6x)
variant 6 series	ACTGGGCTTTTGCTTCATTTGTTCAG (exon 1x/y/z)	AGAGAATTCTTCCAGGTGGCAAGG (exon 6/6x)
	CGACTGGAGCACGAGGACACTGA (5^′^ RACE)	ACGAGTGAAGCAACATGGCACAAAG (exon 1x/y/z)
	ACTGGGCTTTTGCTTCATTTGTTCAG (exon 1x/y/z)	GCTGTCAACGATACGCTACGTAACG (3^′^ RACE)
	AGGAGTCTTTGTGCCATGTTGCTTCAC (exon 1x/y/z)	CGCTACGTAACGGCATGACAGTG (3^′^ RACE)
	GGACACTGACATGGACTGAAGGAGTA (5^′^ RACE)	ACTCCTGAACAAATGAAGCAAAAGCCCC (exon 1x/y/z)
ACTB	ATTCCTATGTGGGCGACGAGGC (exon 3)	TGGATAGCAACGTACATGGCTGG (exon 4)
Quantitative RT-PCR		
NM_001175	GGACAGAGACGTGAAGCACTGA (exon 1)	GCTTGCTGTCCAGCTCATCA (exon 2)
variant 6a	GTCTTTGTGCCATGTTGCTTCA (exon 1x/y/z)	AGCTTGCTGTCCAGCTCATCA (exon 2)
ACTB	TGGCACCCAGCACAATGA (exon 5)	CCGATCCACACGGAGTACTTG (exon 6/6x)
Standard curves in quantitative RT-PCR		
NM_001175	ACTCAGAAGTCAGAGTTGAGAGAC (exon 1)	AGAGAATTCTTCCAGGTGGCAAGG (exon 6/6x)
variant 6a	ACTGGGCTTTTGCTTCATTTGTTCAG (exon 1x/y/z)	ACTCTCACAAACCAGGGTGAGC (exon 3)

### Rapid amplification of cDNA ends and DNA sequencing analysis

**Figure 1:**
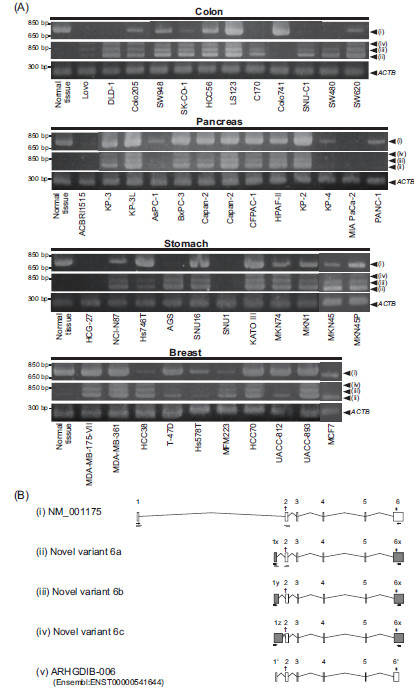
***ARHGDIB *expression pattern in cell lines and normal tissues.** (**A**) *ARHGDIB* expression profile in colon, pancreas, stomach, and breast cancer cell lines. Amplicons (i), (ii), (iii), and (iv) correspond to the 4 types of transcripts shown in Panel **B**. (**B**) Exon structures on the *ARHGDIB* gene. The 5 forms shown include the known transcript NM_001175 (i), 3 novel variants (ii–iv), and the known variant ARHGDIB-006 predicted in Ensembl (v). The grey boxes show novel alternative splicing forms in variant 6a (ii), 6b (iii), and 6c (iv). The † and * symbols indicate the locations of the start codon and the stop codon, respectively. The number of exons is shown on the white and grey boxes. Primer positions for RT-PCR and qRT-PCR are indicated by black and grey arrows, respectively.

To determine the sequence of novel ARHGDIB transcripts, rapid amplification of cDNA ends (RACE) was conducted using the GeneRacer Kit (Invitrogen) according to the manufacturer’s instructions. The primer sequences are shown in Table 1. For DNA sequencing analysis, the PCR products were analysed on 1–2% agarose gels by electrophoresis following by gel staining with SYBR Safe (Invitrogen). The bands visualized under ultraviolet light were isolated and purified using the QIAquick Gel Extraction Kit (Qiagen). The purified samples were then cloned into a pCR 2.1-TOPO vector (Invitrogen) using the TOPO TA Cloning Kit for Sequencing (Invitrogen). Positive transformants were sequenced using the Big Dye Terminator v3.1 Cycle Sequencing Kit (Applied Biosystems, Foster City, CA, USA) and an ABI 3130xl Genetic Analyzer (Applied Biosystems). The start and stop codons were determined from the identified sequences and are shown in Figure 1B.

### Quantitative real-time PCR

Quantitative real-time PCR (qRT-PCR) was performed using the SYBR Green dye technique and the ABI PRISM 7900HT Fast Real Time PCR System (Applied Biosystems). The target fragment among the synthesized cDNA was amplified using specific primers (Table 1) according to the following protocol: preheating at 95°C for 20 sec; 40 cycles at 95°C for 1 sec and 60°C for 30 sec; and dissociation at 95°C for 15 sec, 60°C for 15 sec, and 95°C for 15 sec. The threshold cycle (Ct) values were converted into absolute copy numbers using a standard curve. This standard curve was constructed using data for purified PCR amplicons that were generated from the plasmids containing the sequences of the target transcripts. These conversions for quantification were performed for data obtained at high PCR amplification efficiencies (95–105%). Nonspecific amplification in qRT-PCR was checked by dissociation curve analysis and gel electrophoresis, which showed the amplicon size.

### Immunoblotting

SW948, Colo741, and DLD-1 cells were harvested using a trypsin–EDTA solution (Invitrogen) and washed twice with phosphate-buffered solution (PBS, pH 7.4). After removal of the supernatant, cellular pellets were ultrasonicated in PBS with a protease inhibitor cocktail (Roche Diagnostics, Basel, Switzerland). Centrifugation of the lysates at 15,000 *g* for 10 min was followed by collection of the supernatant for determination of protein concentration using the Quick Start Bradford Protein Assay Kit (Bio-Rad, CA, USA). The quantified lysates were then subjected to one-dimensional sodium dodecyl sulphate-polyacrylamide gel electrophoresis (SDS-PAGE) using 5 μg of lysate protein/well on 12.5% polyacrylamide gel. After electroblotting of the lysate onto polyvinylidene difluoride membranes (pore size, 0.45 μm; Millipore, Bedford, MA, USA), the blots were probed with a mouse monoclonal anti-ARHGDIB/D4-GDI antibody (1:500 dilution; Acris Antibodies GmbH, Herford, Germany) and a rabbit polyclonal anti-TUBA4A antibody (1:5,000 dilution; Santa Cruz Biotechnology, Santa Cruz, CA, USA). The bound antibodies were incubated with the corresponding anti-IgG secondary antibodies conjugated with horseradish peroxidase (1:50,000 dilution; Jackson Laboratories, Bar Harbor, ME, USA). Specific proteins were visualized using the ECL Plus Western Blotting Detection System (GE Healthcare, Little Chalfont Buckinghamshire, England) and the FUJIFILM Luminescent Image Analyzer LAS3000 (Fuji Film, Tokyo, Japan).

### mRNA stability assay

To assay mRNA stability, actinomycin D was added to the culture medium when the SW948 cells reached 80–90% confluence. During the following 24 h, cells were harvested at 0, 2, 4, 6, 8, 10, or 24 h, and total RNA was extracted as described above. The amount of target transcripts (known form of ARHGDIB and variant 6a) and actin β (ACTB) at each time point was quantified using qRT-PCR.

## Results

### Identification of novel ARHGDIB exons

ARHGDIB is known to be involved in the progression of metastasis [10,12,14-17] in colon, pancreas, stomach, and breast cancer. Therefore, systematic RT-PCR screening of 46 cancer cell lines and of normal colon, pancreas, stomach, breast, and placental tissue was conducted using newly designed RT-PCR primers for several exons on the target gene and a primer pair spanning the region from exon 1 to exon 6 on the known ARHGDIB transcript registered in GenBank (NM_001175; Figure 1A). RT-PCR screening revealed positive signals (i) in 36 cell lines (78%) and all 4 types of normal tissue whose lengths were identical to those of the known transcript. The RT-PCR subsequently performed using another primer pair designed for annealing within exon 1 of the variant ARHGDIB-006 (ENST00000541644), which is predicted in the Ensembl and Havana models (http://www.ensemb.org), did not result in detection of the expected band. Likewise, other splice forms whose detection had been anticipated were not observed. Although other bands (ii–iv) were observed in 35 cell lines (76%), these bands were not detected in all the normal tissues. These results indicate that the unknown *ARHGDIB* transcripts are expressed along with the known ubiquitously expressed *ARHGDIB* transcripts only in cancer cells.

To determine the exon structures of the unknown transcripts (ii-iv), designated as variants 6a, 6b, and 6c, respectively (Figure 1B), DNA sequencing was performed using RACE. Although the spliced forms were found to consist of 6 exons, as seen in the predicted variant (ARHGDIB-006), their first and last exons differed from those of the predicted variant (Figure 1B). Specifically, the first exons (exon 1x–z) were derived from the intronic region in the known transcript and the sixth exons (exon 6x) from a part of exon 6 identical to the known transcript, while exons 2 to 5 were common in all the novel transcripts. Although the exon structures of these novel and known variants, along with the known transcript, were found to vary, no differences were observed in their protein-coding regions.

### ARHGDIB splice variants in normal tissues

**Figure 2:**
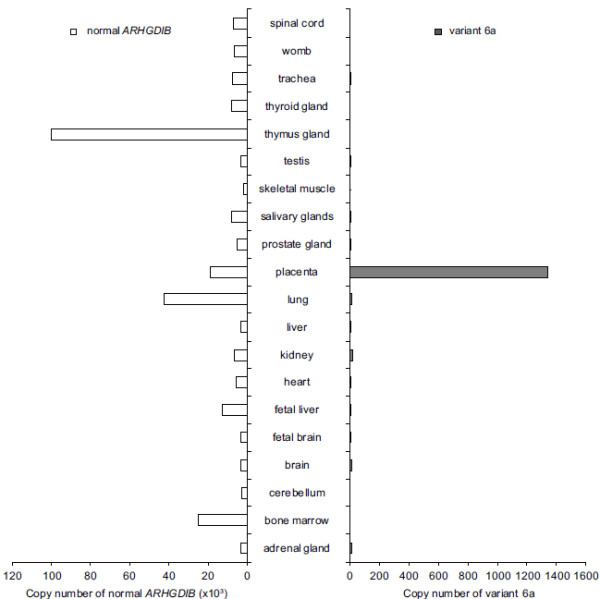
**Comparison of splicing events in normal tissues.** The copy number of transcripts was estimated by determining the mean of the values obtained by qRT-PCR analysis performed in duplicate. The left and right panels show the expression patterns of normal ARHGDIB and variant 6a, respectively.

Novel splice variants were detected in colon, pancreas, stomach, and breast cancer cells but not in the corresponding normal tissues. To verify the cancer specificity of these variants, their mRNA expression profile in other normal tissues was investigated using qRT-PCR (Figure 2). As shown in Figure 1A, all 3 variants were expressed in most of the cancer cell lines (6a–c), which appeared to express variant 6a at the highest level. The mRNA expression level of variant 6a was examined on the basis of these results. Normal *ARHGDIB* expression was found to be ubiquitous in the normal tissues analysed, consistent with previously reported results [11]. In contrast, mRNA expression of variant 6a was only detected in placental tissue, while the amount of variant 6a transcript in the other normal tissues was below the detection limit of qRT-PCR. These results indicate that the cancer-specific splice variants identified in this study are also expressed in normal placental tissue.

### Validation of ARHGDIB mRNA and protein expression levels

**Figure 3:**
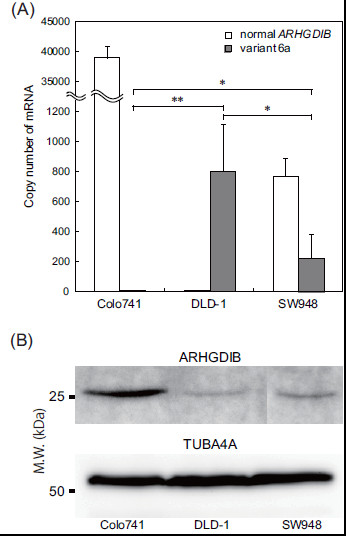
**Expression analysis of ARHGDIB at the mRNA and protein levels in colon cancer cell lines.** (**A**) The expression levels of known *ARHGDIB* transcripts and variant 6a transcripts were analysed by qRT-PCR. (**B**) Protein expression was confirmed by immunoblotting with the ARHGDIB-specific monoclonal antibody. Immunoblotting of tubulin, α 4a (TUBA4A) protein, was conducted as a positive control experiment. The colon cancer cell lines Colo741, DLD-1, and SW948 were examined as representative cancer cell lines whose expression profiles of variant 6a differs from that of the normal transcript. Data are expressed as mean ± SD values calculated from information obtained from an experiment repeated 4 times. Statistical significance (*p < 0.05 and **p < 0.01) was evaluated by Student’s *t-*test.

Despite significant differences in the 5^′^ and 3^′^ untranslated regions (UTRs) between the known *ARHGDIB* transcript and the variant 6 series (6a, 6b, and 6c), the sequences of their protein coding regions were found to be identical in all the transcripts examined. Validation of mRNA and protein expression levels was conducted using Colo741, DLD-1, and SW948, colon cancer cell lines that have shown discriminating patterns of mRNA expression (Figure 1A). Specifically, Colo741 has been found to highly express the known transcript, DLD-1 has been found to express only the novel variants, and SW948 has been found to express both transcripts. To quantify their mRNA levels more precisely, qRT-PCR analysis was first performed. The mRNA level of variant 6a was reflected in the visualized image of the RT-PCR amplicons (Figure 3A), and the expression patterns of variants 6b and 6c in the 3 cell lines were found to be similar to that of variant 6a (data not shown). Immunoblotting performed to clarify the ARHGDIB protein level led to recognition of the amino acid sequence positioned between 125 and 137 in the target protein by the ARHGDIB antibody used in this study (Figure 3B). As the protein-coding region of the identified variants was found to be identical to that of the normal *ARHGDIB* transcript, the stained protein in the immunoblots was considered to have been translated from both the normal and variant RNA. Nevertheless, the expression levels of this protein in the 3 cell lines were proportional to those of the mRNA levels of the normal transcript. Although the mRNA expression of variant 6a was higher in DLD-1 than in SW948, the ARHGDIB protein expression levels in both cell lines were inversely correlated with the transcript levels. These results raise the possibility that the translation of variant 6a into intact protein is partially suppressed through the alteration of exon structures.

### Stability of variant 6a mRNA

**Figure 4:**
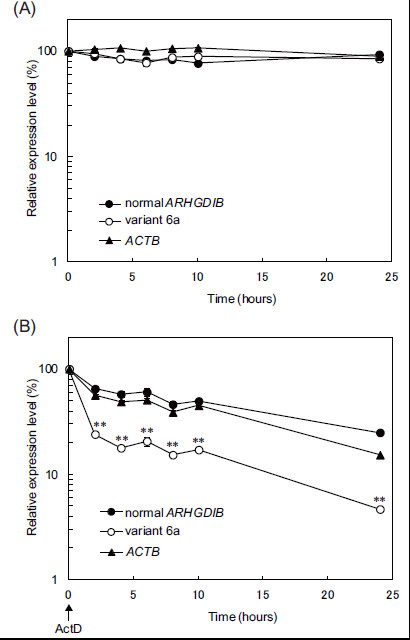
**Stability of ARHGDIB variant 6a mRNA and normal ARHGDIB mRNA.** SW948 cells were cultured without (**A**) or with (**B**) actinomycin D (ActD). Amplification of actin β (*ACTB*) was conducted as a positive control experiment. The expression levels of transcripts before addition of ActD are shown as 100%. Data are expressed as mean ± SD values calculated from information obtained from an experiment repeated 4 times. Statistical significance (**p < 0.01) was evaluated by Student’s *t-*test.

The stability of mRNA, which regulates protein expression, is affected by UTR variation through alternative splicing [19,20]. To evaluate the mRNA stability of variant 6a, the half-life of normal *ARHGDIB* mRNA and that of variant 6a mRNA was determined in SW948 cells that equally expressed both forms of mRNA. To inhibit transcription, SW948 cells were treated with actinomycin D, while amplification of ACTB was conducted as a positive control experiment. The relative expression levels of normal *ARHGDIB* mRNA and variant 6a mRNA in SW948 cells treated without actinomycin D remained stable during 24 h of observation (Figure 4A), and their half-life values were estimated to be 8 h and 1 h, respectively (Figure 4B). These results suggest that the mRNA stability of variant 6a is lower than that of normal *ARHGDIB*.

## Discussion

Alternative splicing, a phenomenon by which a single gene generates multiple forms of mRNA that can be translated into diverse proteins [21], is responsible for an estimated 10–20% of all cancer-related gene alterations [5,22,23]. Both the ubiquitous normal transcripts and the unique splice variants generated from these alteration events are frequently expressed simultaneously in cancerous cells and tissues [6,7,24]. As such, those variants expressed as cancer-specific transcripts are possible candidate biomarkers [6,7]. This study revealed that while the splice variants 6a, 6b, and 6c of the *ARHGDIB* gene are expressed in pancreas, stomach, colon, and breast cancer cell lines and in normal placental tissue but not in other types of normal tissue, the known form of *ARHGDIB* is ubiquitously expressed in many types of normal tissue and cancer cells. A similarly restricted profile has also been observed for PLAC1. PLAC1, reported to be the first member of a new class of antigens that specifically relates placentation to cancer [25,26], has been observed to be ectopically activated in many human cancers and is essentially involved in malignant cell processes [25]. These *ARHGDIB* variants can be considered potential biomarkers based on the similarity between ARHGDIB and PLAC1 reported in previous studies, as well as the observation of high levels of expression of the variants 6a, 6b, and 6c in placental and cancer cells and their relatively restricted expression in normal tissues in this study.

Splice variants are often translated into structurally defective proteins in cancer cells, affecting the functioning of the original protein [7,27]. In a previous study, the authors of this study focused on examining splice variants and variant-derived protein isoforms to identify candidate biomarkers [6]. However, the current study revealed that the variants 6a, 6b, and 6c and the normal transcript share a common protein-coding region and that these variants are slightly translated into the intact ARHGDIB protein. These results suggest that the structurally defective protein isoforms of ARHGDIB derived from the variant 6 series are not expressed in cancer cells and the placenta, thereby indicating that the mRNA of the cancer-specific variant 6 series may be a more suitable biomarker than the ARHGDIB protein.

In general, mRNA stability is related to UTR length as well as secondary structure [20], as both influence the regulation of protein expression [19]. In this study, the 5^′^ and 3^′^ UTRs of the variant 6 series were observed to clearly differ from those of the normal ARHGDIB, resulting in the decreased mRNA stability of these variants, while the translation efficiency of the variants into intact ARHGDIB protein was observed to be distinctly low. These findings suggest that the mRNA stability of the novel variants identified in this study may be involved in the regulation of protein expression.

## Conclusions

In summary, validation analysis using RT-PCR and DNA sequencing successfully identified variants 6a, 6b, and 6c—3 novel splice variants of the *ARHGDIB* gene—in colon, pancreas, stomach, and breast cancer cell lines; these variants were also found to be highly expressed in normal placental tissue. In contrast, the mRNA expression of known *ARHGDIB* was ubiquitous in many normal tissues and cancer cells. These findings suggest that these cancer and placenta-specific variants possess cancer-specific functions and, as such, are potential cancer-related biomarkers.

## Competing interests

The authors declare that they have no competing interests.

## Authors’ contributions

KH and YF designed the study, interpreted the data, and drafted the manuscript. KO assisted in drafting the manuscript. KO, MT, KY, and TM were scientific leads and assisted in designing the study. MT, KY, and TM provided reagents, materials, and analysis tools. All authors read and approved the final manuscript.

## Supplementary Material

Additional file 1 List of all cell lines and normal tissues examined. Click here for file
